# TiO_2_ Nanowires with Doped g-C_3_N_4_ Nanoparticles for Enhanced H_2_ Production and Photodegradation of Pollutants

**DOI:** 10.3390/nano11010254

**Published:** 2021-01-19

**Authors:** Liushan Jiang, Fanshan Zeng, Rong Zhong, Yu Xie, Jianli Wang, Hao Ye, Yun Ling, Ruobin Guo, Jinsheng Zhao, Shiqian Li, Yuying Hu

**Affiliations:** 1 College of Environment and Chemical Engineering, Nanchang Hangkong University, Nanchang 330063, China; jiangliushan@126.com (L.J.); zengfanshan@126.com (F.Z.); zhongrong@126.com (R.Z.); wangjianli@126.com (J.W.); yehao@126.com (H.Y.); lingyun@126.com (Y.L.); guoruobin@126.com (R.G.); 2 Shandong Key Laboratory of Chemical Energy Storage and Novel Cell Technology, Liaocheng University, Liaocheng 252059, China; 3 School of Ocean Science and Biochemistry Engineering, Fuqing Branch of Fujian Normal University, Fuqing 350300, China; lishiqian@126.com; 4 School of Civil Engineering and Architecture, East China Jiaotong University, Nanchang 330013, China

**Keywords:** TiO_2_ nanowire, g-C_3_N_4_ particles, g-C_3_N_4_/TiO_2_ composite, photodegradation of pollutants

## Abstract

With the rapid consumption of fossil fuels, along with the ever-increasing environmental pollution, it is becoming a top priority to explore efficient photocatalysts for the production of renewable hydrogen and degradation of pollutants. Here, we fabricated a composite of g-C_3_N_4_/TiO_2_ via an in situ growth method under the conditions of high-temperature calcination. In this method, TiO_2_ nanowires with a large specific surface area could provide enough space for loading more g-C_3_N_4_ nanoparticles to obtain C_3_N_4_/TiO_2_ composites. Of note, the g-C_3_N_4_/TiO_2_ composite could effectively photocatalyze both the degradation of several pollutants and production of hydrogen, both of which are essential for environmental governance. Combining multiple characterizations and experiments, we found that the heterojunction constructed by the TiO_2_ and g-C_3_N_4_ could increase the photocatalytic ability of materials by prompting the separation of photogenerated carriers. Furthermore, the photocatalytic mechanism of the g-C_3_N_4_/TiO_2_ composite was also clarified in detail.

## 1. Introduction

The vigorous development of various industries drives rapid economic progress but causes excessive energy consumption and environmental pollution [1]. Such problems should not be underestimated; thus, finding efficient technology and sustainable energy has become the top priority. In recent years, hydrogen energy (H_2_) has been greatly popular with scientists because of its peculiarities of being renewable and clean. Considerable efforts have been devoted to the development of advanced technologies for harvesting hydrogen, and water splitting catalyzed by semiconductors is widely recognized as a promising approach to producing H_2_ [2,3].

TiO_2_ is a robust photocatalyst for water splitting for generating hydrogen, photodegradation, dye-sensitized solar cell biosensors, and other fields due to its high photostability, outstanding chemical stability, nontoxicity, and high efficiency [4,5]. However, the applications of TiO_2_ are still greatly limited by its intrinsic shortcomings. For example, the wide band gap (3.2 eV) and single crystalline phase can reduce the migration efficiency and lead to a high recombination rate for charges or limit the utilization of solar energy [6,7,8]. Therefore, numerous scientists have devoted themselves to solving these difficulties from different perspectives, such as the auto-doping of TiO_2_ [9], noble metal deposition, heterojunction construction, and ion doping [10,11,12,13,14,15,16,17]. Among these methods, the construction of heterojunctions is believed to be one favorable way [18].

Over the past decade, g-C_3_N_4_ has drawn worldwide attention due to its advantages of high stability, being green, being cheap, and being easy to synthesize, as well as unique electronic structure [19,20]. Furthermore, the morphology of materials also impacts the performance. The surface area of nanowires is dozens of times bigger than that of particles and has a strong absorption capacity. Meanwhile, nanowires can be filled with other nanomaterials to generate nanocomposites for greatly enhancing photocatalytic ability [21].

Herein, we propose a strategy of combining TiO_2_ nanowires and g-C_3_N_4_ nanoparticles for generating a nanocomposite, aiming at improving photocatalytic performance. Considering that the synthetic process should be environmentally friendly and low cost as well as efficient, we used commercial P25 powder and sodium hydroxide (NaOH) as raw materials to prepare TiO_2_ nanowires. Under the reaction conditions of high temperature, high pressure, and strong alkalinity, P25 reacted with NaOH to produce titanium, which convolved to form a tubular structure after the Na^+^ was exchanged in the washing process. Simultaneously, hydroxide was dissolved in the water, and oxide precipitated due to the different solubility. At last, g-C_3_N_4_ nanoparticles were loaded on the TiO_2_ nanowires to construct a novel g-C_3_N_4_/TiO_2_ heterojunction via an in situ growth method at a high calcination temperature. Furthermore, the photocatalytic ability of all the materials was examined by the photodegradation of pollutants and photocatalytic hydrogen production.

## 2. Experimental Section

### 2.1. Experimental Reagents

Titanium dioxide (TiO_2_, P25, nanoscale) and sodium hydroxide (NaOH, AR) were both obtained from Xilong Chemical Co., Ltd (Shantou China). Melamine (C_3_N_3_(NH_2_)_3_) was purchased from Sinopharm Chemical Reagent Co., Ltd (Shanghai, China).

### 2.2. Synthesis of TiO_2_ Nanowires

The TiO_2_ nanowires were prepared according to a previous report with some modifications [22]. Firstly, P25 (0.5 g) was dispersed into a 75 mL NaOH solution (10 mg/L) using an ultrasonic instrument for 30 min, and then was stirred for another 30 min. After that, the well-mixed reactant was poured into a Teflon reactor (100 mL) and reacted at 130 °C for 24 h. After cooling down to room temperature, the precursor was obtained through centrifugation and washed until the pH was 9. Afterwards, the above product was washed and stirred in 100 mL of HNO_3_ (0.1 mol/L) for 5 h. After pickling, the solution was purified with deionized water until the pH was 7 and centrifuged to obtain precipitates. The precipitate was dried in a vacuum at 60 °C for 24 h and then calcined at 600 °C for 3 h with a heating rate of 5 °C/min to obtain TiO_2_ nanowires.

### 2.3. The Preparation of g-C_3_N_4_/TiO_2_ Samples

g-C_3_N_4_ was prepared by calcining the melamine at 500 °C. The as-prepared TiO_2_ nanowire powder and 1 g of melamine were evenly ground, and then calcined at 540 °C for 4 h with a heating rate of 3 °C/min. The different mass ratios of g-C_3_N_4_/TiO_2_ composite (such as 20%, 30%, 40%, and 50%) were synthesized by changing the quality of g-C_3_N_4_ without the other conditions being changed.

### 2.4. Characterization

The phase and textual properties of the samples were tested by X-ray powder diffraction (XRD). The micromorphology and lattice structure were further analyzed with a scanning electron microscope (SEM) and transmission electron microscope (TEM). The chemical compositions and states were determined by X-ray photoelectron spectroscopy (XPS). Besides, the surface area and porosity were determined on a specific surface area tester. The UV-vis diffuse reflection spectra (UV-vis DRSs) of the samples were acquired with an ultraviolet and visible spectrophotometer with an integrating sphere; BaSO_4_ was used as the background material. The photocurrent tests were carried out with an electrochemical workstation (CHI 660D) with a standard three-electrode system.

### 2.5. The Photocatalytic Ability Tests

#### 2.5.1. The Experiments for Photodegradation

The photodegradation tests of methyl orange and rose red were carried out under a 300 W xenon light with a light filter (λ > 420 nm). Firstly, 50 mg of as-prepared g-C_3_N_4_/TiO_2_ complex was ultrasonically dispersed in a beaker containing the pollutant (50 mL, 10 ppm) and then magnetically stirred in the dark for 60 min to achieve the absorption–desorption equilibrium, labeled C_0_. Afterwards, the treated solution was irradiated under the xenon light for 120 min to perform the photodegrade reaction. Then, 3 mL of the pollutant suspension was extracted at the given interval times and then centrifuged and labeled C_t_. At last, the change in the concentration of the contaminant was measured with an ultraviolet–visible spectrophotometer.

#### 2.5.2. Photocatalytic H_2_ Generation

The photocatalytic hydrogen evolution reaction was performed in a quartz glass at 279.15 K. Meanwhile, the quartz glass was connected with a closed circulation system, and a 300W xenon light served as the light source. Typically, g-C_3_N_4_/TiO_2_ complex (50 mg) was dispersed in the solution, which consisted of methyl alcohol (8 mL) and deionized water (72 mL); then, Ar was pumped into the system to remove the air. Afterwards, the above reactor was illuminated under a 300 W xenon light for 3 h. At intervals of 0.5 h, the generated hydrogen gas was quantified with a gas chromatograph (GC7900).

#### 2.5.3. The Tests for Photocurrent

The photocurrent experiments for the g-C_3_N_4_/TiO_2_ composite were monitored with an electrochemical station (CHI660C) with 0.5 mol/L NaSO_4_ solutions as the electrolyte solution. For this, the Ag/AgCl electrode was used as the reference electrode, and the graphite electrode and prepared samples served as the auxiliary electrode and the working electrode, respectively. Typically, the working electrode could be fabricated by the following method: 2 mg samples were evenly dispersed in 1 mL of deionized water. Then, 100 μL of suspension liquid was coated uniformly on tin oxide conductive glass, dried at room temperature, and set aside.

## 3. Results and Discussion

### 3.1. Micromorphology and Lattice Structural Characteristics

**Figure 1:**
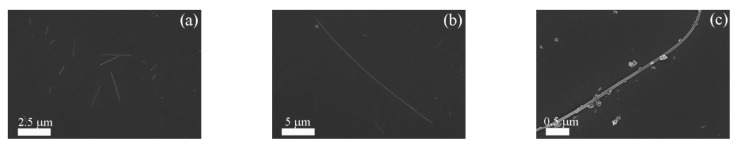
SEM images of TiO_2_ (**a**,**b**) and g-C_3_N_4_/TiO_2_ composite (**c**).

The micromorphology of the samples was preliminarily studied through the SEM images (Figure 1). Obviously, the shape of TiO_2_ was wirelike with a smooth surface and uniform thickness (Figure 1a,b). Meanwhile, it can be seen that the length of the TiO_2_ nanowires was inhomogeneous. This was attributed to the fact that P25 reacted with NaOH, forming titanate, which was convolved to a linear structure after sodium ions were exchanged in the washing process. Besides, the reason for the different lengths may also have been the high-temperature calcination. As shown in Figure 1c, we can clearly observe that the g-C_3_N_4_ particles stuck tightly on the TiO_2_ nanowires. The longer TiO_2_ nanowires were able to provide sufficient supporting space for the in situ growth of g-C_3_N_4_, which made it possible to load g-C_3_N_4_ on the TiO_2_.

**Figure 2:**
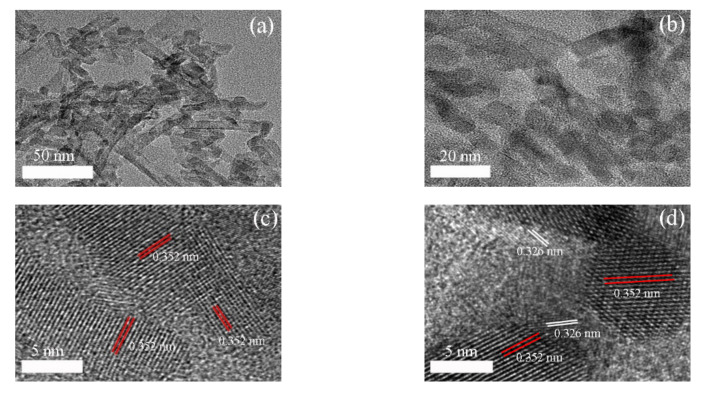
TEM images of TiO_2_ (**a**) and g-C_3_N_4_/TiO_2_ (**b**); HRTEM images of TiO_2_ (**c**) and g-C_3_N_4_/TiO_2_ (**d**).

In addition, we used a transmission electron microscope (TEM) to further view the morphology, particle size, and lattice structure of the samples. Similarly, the morphology (Figure 2a,b) of the TiO_2_ and g-C_3_N_4_ was nanowires and particles, respectively. These was consistent with the SEM images. The lattice fringes at a distance of 0.352 nm (Figure 2c,d) corresponded to the (101) plane of TiO_2_ [22,23,24], while the lattice fringes at 0.326 nm (Figure 2d) could be assigned to the crystal plane of g-C_3_N_4_ [25,26]. Therefore, it could be identified that the g-C_3_N_4_/TiO_2_ was successfully synthesized. In addition, the crossed and overlapped lattice fringes of the partial g-C_3_N_4_ and TiO_2_ reflected the idea that the coupling of g-C_3_N_4_ and TiO_2_ was not simple surface contact but the presence of chemical bonding. During the reaction process, Ti(OH)_4_ particles of the TiO_2_ nanowires were in contact with the triazine structure in the melamine, and then, the precursor was polymerized with high-temperature calcination. Therefore, TiO_2_ nanowires closely contacted g-C_3_N_4_ via chemical bonds, thereby generating a heterojunction.

### 3.2. Crystal Phase and Textural Characteristics

**Figure 3:**
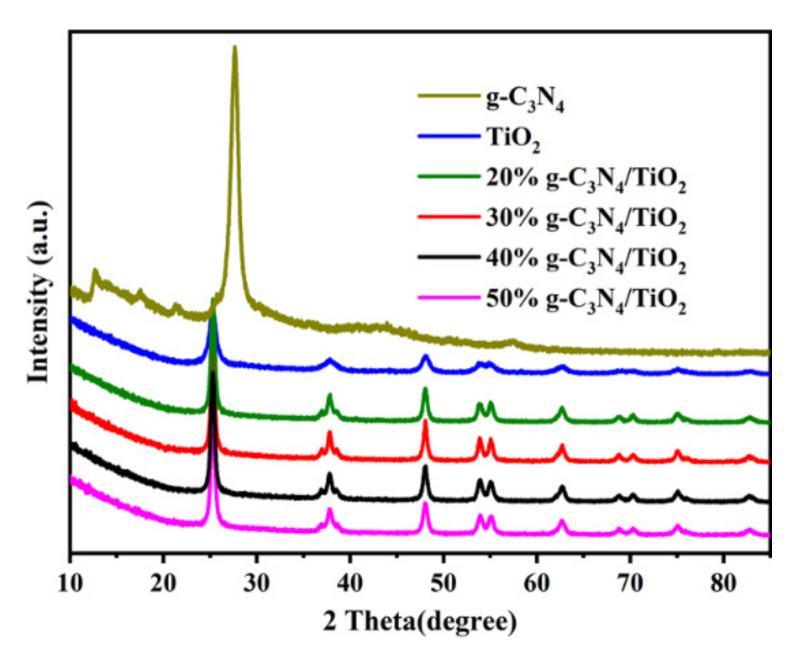
The XRD patterns of TiO_2_, g-C_3_N_4_, and g-C_3_N_4_/TiO_2_ samples with different mass ratios.

The crystal form and crystallinity of the samples were analyzed by obtaining the X-ray diffraction (XRD) patterns. The XRD patterns of the materials displayed five strong diffraction peaks located at 25.2°, 37.7°, 47.7°, 53.9°, and 54.5° (Figure 3), which corresponded to the (101), (004), (200), (105), and (211) crystal planes of TiO_2_ (ICPDS card No.21-1272), respectively [27]. This result indicates the as-prepared TiO_2_ was anatase phase. For g-C_3_N_4_, the characteristic peaks at 13.1° and 27.4° were consistent with the standard card for g-C_3_N_4_ [28]. However, no distinct characteristic peak of g-C_3_N_4_ was observed on the XRD patterns of the composite, probably due to the low content of g-C_3_N_4_ [29].

**Figure 4:**
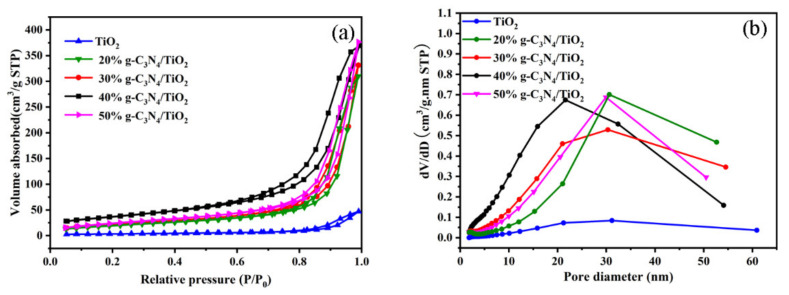
The N_2_ adsorption–desorption isothermal curve (**a**); pore diameter distribution curves (**b**).

In general, the pore characters and specific surface areas of materials have great influences on the photocatalytic performance of the materials. The N_2_ absorption–desorption isotherms and pore diameter distribution are shown in Figure 4. As shown in Figure 4a,b, the lack of hysteresis loops and lack of obvious pore size distribution peaks in the TiO_2_ curve indicate that there was no large pore structure or mesoporous structure [30]. With an increase in g-C_3_N_4_ loading content, we found that curves typical of 20% g-C_3_N_4_/TiO_2_, 30% g-C_3_N_4_/TiO_2_, 40% g-C_3_N_4_/TiO_2_, and 50% g-C_3_N_4_/TiO_2_ belonged to the type-IV absorption isotherm, with a H_3_ hysteresis loop at higher pressures (0.8–1.0), which clarified the presence of mesoporous structure in the samples [31,32]. The formation of the pore was on account of the accumulation of g-C_3_N_4_ nanoparticles on the surface of the TiO_2_ nanowires, and the g-C_3_N_4_ changed the surface roughness of the nanowires’ structure. As shown in Table 1, with the loading of g-C_3_N_4_ increasing from 20% to 50%, the specific surface areas of the samples firstly increased and then decreased, but the pore volume almost remained unchanged. This is because the excessive loading of g-C_3_N_4_ led to the aggregation of the g-C_3_N_4_ nanoparticles and thus reduced the specific surface area of the TiO_2_ nanowires [20].

### 3.3. Chemical State and Band Gap Analysis

**Figure 5:**
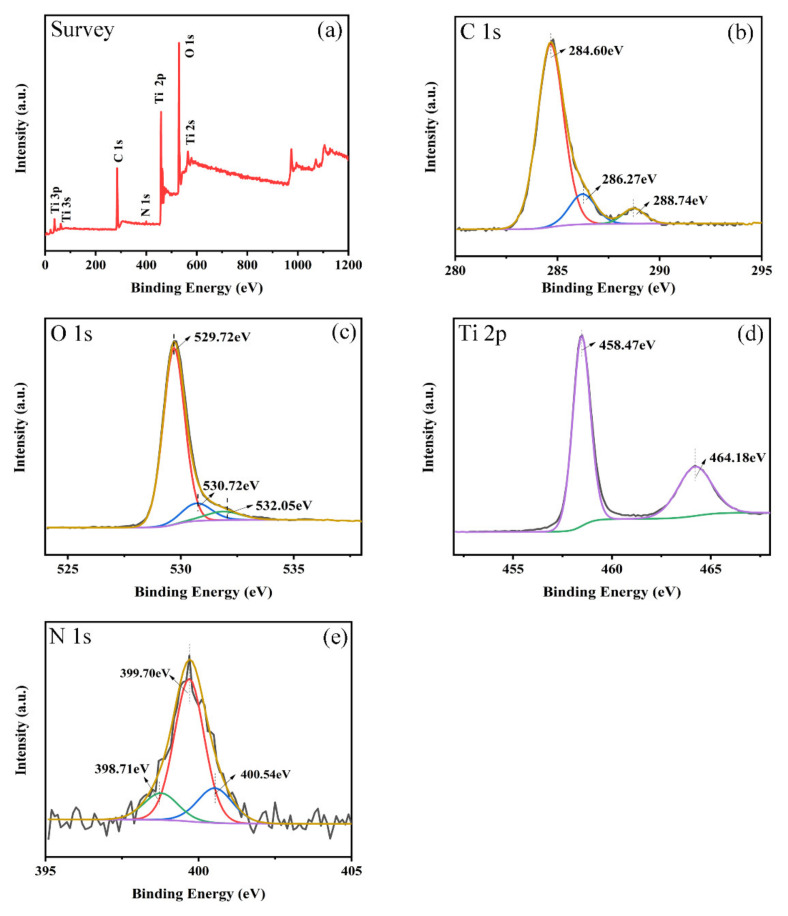
The high-resolution XPS spectra of the 40% g-C_3_N_4_/TiO_2_ composite: survey (**a**), C 1s (**b**), O 1s (**c**), Ti 2p (**d**), and N 1s (**e**).

Typically, XPS spectra are a powerful method for investigating the electronic structures of different elements in nanocomposites. The characteristic peaks of Ti, O, and C were detected in the survey of the g-C_3_N_4_/TiO_2_ composite (Figure 5a), which suggested the successful synthesis of the nanocomposite containing g-C_3_N_4_ and TiO_2_. The XPS spectra of C 1s (Figure 5b) displayed three peaks located at 284.6, 286.27, and 288.74 eV, corresponding to C-C, C-OH, and C=O (and COO) bonding, respectively [20,33]. In the case of O 1s (Figure 5c), the binding energies of 529.72, 530.72, and 532.05 eV were attributed to Ti-O, H-O, and C-O, respectively [34]. The XPS spectra of Ti 2p displayed two peaks for Ti^4+^ (Figure 5d), indicating that the Ti species were in the form of TiO_2_ [27]. After peak splitting fitting, the broad N 1s peak was divided into three contributions (Figure 5e), namely, C=N-C (398.71 eV), N-C_3_ (399.70 eV), and C-N-H (400.54 eV) [19].

**Figure 6:**
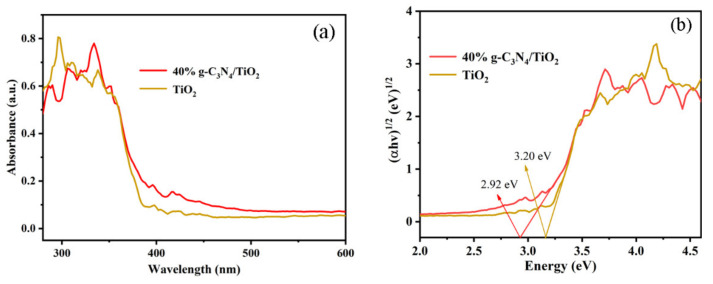
The UV-vis diffuse reflectance spectra (**a**) and band gaps (**b**) of TiO_2_ and g-C_3_N_4_/TiO_2_ composite.

The optical performance of the photocatalysts is shown in Figure 6a. Obviously, the absorption edge of the 40% g-C_3_N_4_/TiO_2_ composite showed a red shift compared with pure TiO_2_, which would be of benefit for enhancing the utilization of solar energy for improving the photocatalytic ability of materials. The band gap value (Figure 6b) of materials can be acquired by plotting (αhν)^1/2^ against hv and then extending the tangent line to intersect the coordinate axis [29]. The band gap value of 40% g-C_3_N_4_/TiO_2_ was lower than that of TiO_2_ and other materials (Figure S3 in Supporting information), which was of benefit for the photocatalytic reaction.

### 3.4. Photocatalytic Ability Analysis

**Figure 7:**
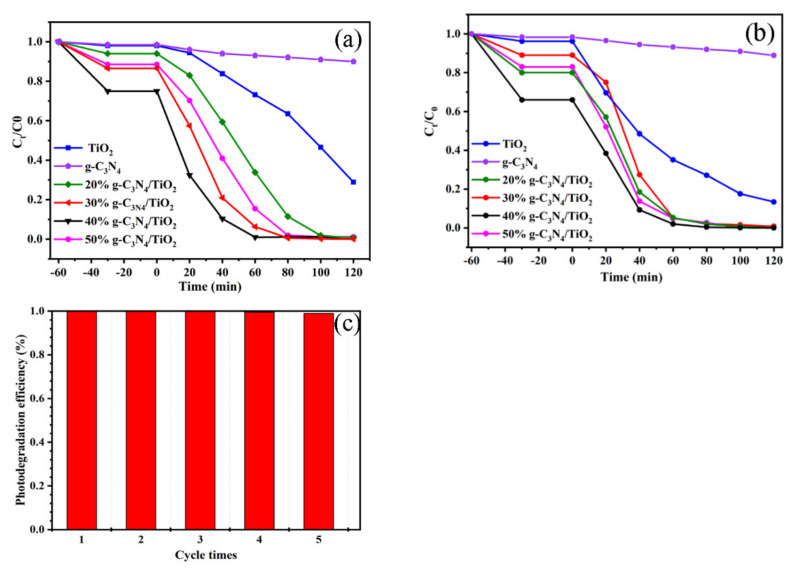
The photodegradation curves for methyl orange (**a**) and the photodegradation curves for rose red (**b**) with g-C_3_N_4_, TiO_2_, and g-C_3_N_4_/TiO_2_ composite; the methyl orange cycling photodegradation experiments with 40% g-C_3_N_4_/TiO_2_ composite (**c**).

During the experimental process, rose red and methyl orange were regarded as the target pollutants for evaluating the photodegradation performance of materials under ultraviolet visible light. As shown in Figure 7a, g-C_3_N_4_ is almost inactive for the photocatalytic degradation of methyl orange. Although the TiO_2_ is active for the degradation of methyl orange, the performance is very poor, with a 70% removal efficiency within 120 min of irradiation time. Impressively, the optimal proportional complex (40% g-C_3_N_4_/TiO_2_) can completely photodegrade the methyl orange in 60 min. Moreover, the efficiency of methyl orange removal by 40% g-C_3_N_4_/TiO_2_ in 20 min was as high as that by TiO_2_ in 120 min. Thus, it can be deduced that the heterojunction in the composite has a positive effect on the photocatalytic performance. Furthermore, the loading content of g-C_3_N_4_ also played a key role in the photodegradation performance. Excessive g-C_3_N_4_ aggregation led to a decrease in the specific surface area and thus limited the reaction. Therefore, we could further deduce that the heterojunction in the complex could inhibit the recombination of carriers facilitating the photocatalytic ability of materials [32].

To further investigate the excellent photodegradation ability of 40% g-C_3_N_4_/TiO_2_ complex, the photodegradation of rose red and methyl orange cycling experiments were also carried out. As shown in Figure 7b, it is obvious that 40% g-C_3_N_4_/TiO_2_ also had a favorable ability to treat rose red. Furthermore, the efficiency of methyl orange degradation by 40% g-C_3_N_4_/TiO_2_ was almost unchanged after five cycling experiments (Figure 7c), indicating that the 40% g-C_3_N_4_/TiO_2_ possessed high stability and reusability. In a word, the above experiments demonstrate that the as-prepared 40%TiO_2_/g-C_3_N_4_ composite has a bright future in applications for wastewater treatment.

**Figure 8:**
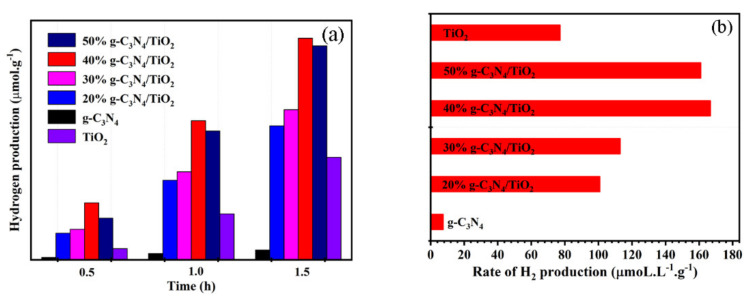
Photocatalytic hydrogen production ability (**a**) and hydrogen production rate tests (**b**) of g-C_3_N_4_, TiO_2_, and g-C_3_N_4_/TiO_2_ samples.

The photodegradation principally made use of the photocatalytic oxidation performance of the photocatalysts, whereas the photocatalytic reduction could also play a part in plenty of fields. Therefore, the photocatalytic hydrogen generation experiments were conducted under ultraviolet–visible irradiation, and the hydrogen produced was quantified every 0.5 h. The H_2_ production performance of pure g-C_3_N_4_ and TiO_2_ was not satisfactory (Figure 8a,b). After coupling g-C_3_N_4_ with TiO_2_, the quantity and rate of hydrogen generation greatly increased, which can be explained as the presence of a heterojunction in the composite. It effectively prompted the separation of photogenerated carriers, thereby improving the performance of hydrogen evolution. However, when the content of g-C_3_N_4_ was 50%, the generation rate for H_2_ became slow because the excessive loading could block the active sites on the TiO_2_.

### 3.5. Photocurrent Analysis

**Figure 9:**
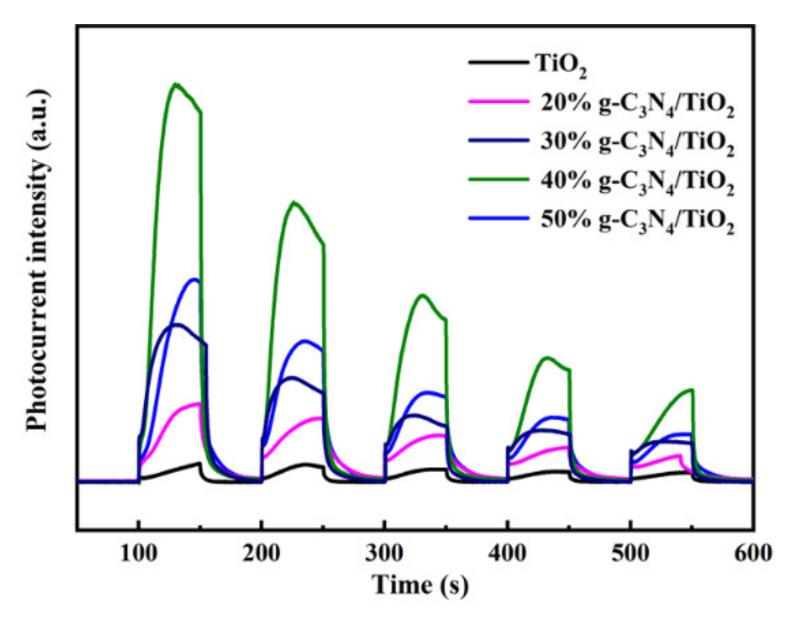
Photocurrent tests of TiO_2_ and g-C_3_N_4_/TiO_2_ samples with different mass ratios.

Usually, the photocurrent response can reflect the transfer and separation of photogenerated charges under irradiation [35]. As depicted in Figure 9, the photocurrent signal of TiO_2_ was the weakest, indicating that the recombination of photogenerated charges was serious. However, the photocurrent response increased after g-C_3_N_4_ was loaded on the surface of the TiO_2_. The reason for this phenomenon was that the heterojunction prompted the separation of photogenerated electrons and holes, thereby providing more electrons that participated in hydrogen evolution [20].

### 3.6. Mechanism of Photocatalysis

**Figure 10:**
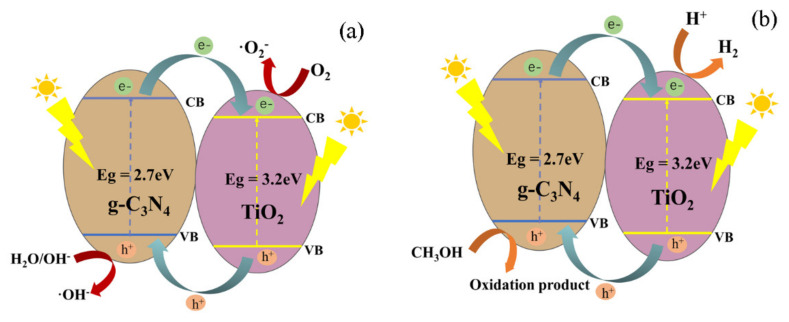
Photocatalytic degradation by (**a**) and hydrogen production (**b**) mechanism of g-C_3_N_4_/TiO_2_ composite.

Figure 10 shows the mechanism of the photodegradation process (a) and the hydrogen production (b). Under irradiation, g-C_3_N_4_ and TiO_2_ were excited to produce a mass of electrons and holes. According to a previous report [36], the conduction band value of g-C_3_N_4_ (−1.3eV vs. NHE) is more negative than that of pure TiO_2_ (−0.5 eV vs. NHE), while the valence band value of TiO_2_ (2.7eV vs. NHE) is more positive than that of g-C_3_N_4_ particles (1.4eV vs. NHE). Therefore, the partial electrons on the conduction band of g-C_3_N_4_ transfer to the conduction band of TiO_2_, enabling O_2_ to change into ·O_2−_. At the same time, the holes in the valence band of TiO_2_ were transferred to g-C_3_N_4_. The OH^-^ and water were oxidated to hydroxyl radicals. At last, the methyl orange and rose red reacted with these radicals to form CO_2_ and H_2_O. Thereby, the electrons and holes were effectively separated. However, the electrons played a major role in the process of hydrogen production. The potential of H_2_ evolution (0 eV vs. NHE) was more positive than the conduction band value of TiO_2_ (−0.5 eV vs. NHE); thus, H^+^ could capture the electrons and change into H_2_.

## 4. Conclusions

In this work, TiO_2_ nanowires were firstly fabricated via the simple hydrothermal method, and then, g-C_3_N_4_ was prepared via thermal polymerization using melamine as the precursor. A novel g-C_3_N_4_/TiO_2_ heterojunction composite was successfully prepared by adjusting the mass ratio of TiO_2_ and melamine in the calcination process. After g-C_3_N_4_ was loaded on the surface of TiO_2_, the absorption range for light, the photocurrent response, and the specific surface area increased significantly, which were benefited by the presence of the heterojunction. The heterojunction in the composite could greatly prompt the separation of the photogenerated electrons and holes, thus enhancing the photocatalytic ability of the g-C_3_N_4_/TiO_2_. More importantly, the g-C_3_N_4_/TiO_2_ composite has the ability to both photocatalyze the degradation of different pollutants and produce hydrogen, which means it has broad application prospects. Meanwhile, the as-prepared sample also kept favorable stability after the cycling experiments. Therefore, this study provides a friendly and effective method for treating complex environmental pollutants and energy consumption.

## Figures and Tables

**Table 1:** The specific surface area of samples.

Samples	Specific Surface Area (m^2^/g)	Pore Volume (cm^3^/g)
TiO_2_	11.97	10.07
20% g-C_3_N_4_/TiO_2_	66.18	10.53
30% g-C_3_N_4_/TiO_2_	80.69	10.51
40% g-C_3_N_4_/TiO_2_	131.18	10.57
50% g-C_3_N_4_/TiO_2_	72.87	10.48

## Data Availability

The data presented in this study are available on request from the corresponding author.

## Supplementary Materials

The following are available online at https://www.mdpi.com/2079-4991/11/1/254/s1. Figure S1: The SEM image of TiO_2_ nanowires, Figure S2: The EDX image of g-C_3_N_4_/TiO_2_ composite. Figure S3: The UV-vis diffuse reflectance spectra (a) and band gaps (b) of 20%g-C_3_N_4_/TiO_2_, 30%g-C_3_N_4_/TiO_2_ and 50% g-C_3_N_4_/TiO_2_ composite.
