# Supplementary material for: TiO2 Nanowires with Doped g-C3N4 Nanoparticles for Enhanced H2 Production and Photodegradation of Pollutants
Source: Nanomaterials (Basel). 2021 Jan 19;11(1):254. doi: 10.3390/nano11010254 (PMC7835803; doi:10.3390/nano11010254)
Supplement: Supplementary file 1 [file nanomaterials-11-00254-s001.pdf]

Supporting Information

Liushan Jiang[1#], Fanshan Zeng[1#], Rong Zhong[1], Yu Xie[1]*, Jiangli Wang[1], Hao Ye[1], Yun Ling[1], Ruobin Guo[1], Jinsheng Zhao[2]*, Shiqian Li[3], Yuying Hu[4]*

[1]College of Environment and Chemical Engineering, Nanchang Hangkong University, Nanchang 330063, PR China;

[2]Shandong Key Laboratory of Chemical Energy Storage and Novel Cell Technology, Liaocheng University, Liaocheng, 252059, PR China;

[3]School of Ocean Science and Biochemistry Engineering, Fuqing Branch of Fujian Normal University, Fuqing 350300, PR China;

[4]School of Civil Engineering and Architecture, East China Jiaotong University, Nanchang 330013, Jiangxi Province, P. R. China

* Corresponding author: Email Address: xieyu_121@163.com (Y. Xie); j.s.zhao@163.com (j.s.zhao); hu_yuying@foxmail.com (Y.Y. Hu)

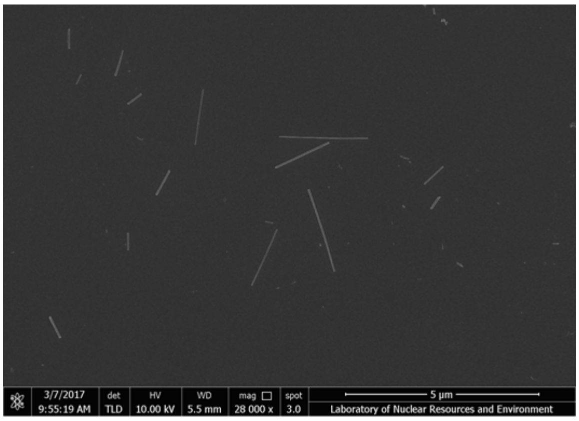

Fig. S1. The SEM image of TiO_2 nanowires.

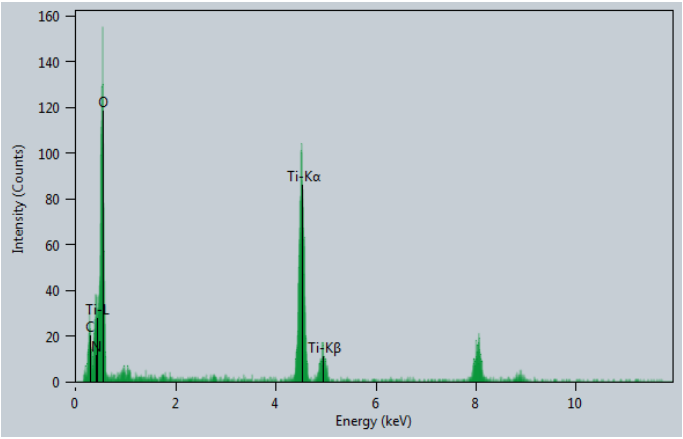

Fig. S2. The EDX image of g-C_3N4/TiO_2 composite.

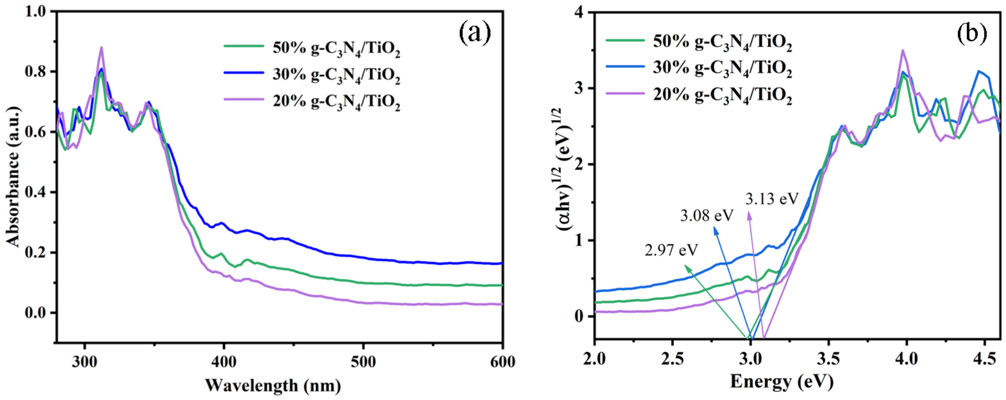

Fig. S3 The UV-vis diffuse reflectance spectra (a) and band gaps (b) of 20%g-C_3N_4/TiO_2, 30%g-$C_3N_4/TiO_2$and 50% g-C_3N_4/TiO_2 composite.
